# Mouse Skull Mean Shape and Shape Robustness Rely on Different Genetic Architectures and Different Loci

**DOI:** 10.3389/fgene.2019.00064

**Published:** 2019-02-12

**Authors:** Ceferino Varón-González, Luisa F. Pallares, Vincent Debat, Nicolas Navarro

**Affiliations:** ^1^Institut de Systématique, Évolution, Biodiversité, ISYEB – UMR 7205 – CNRS, MNHN, UPMC, EPHE, UA, Muséum National d’Histoire Naturelle, Sorbonne Universités, Paris, France; ^2^Biogéosciences, UMR 6282 CNRS, Université Bourgogne Franche-Comté, Dijon, France; ^3^Lewis-Sigler Institute for Integrative Genomics, Princeton University, Princeton, NJ, United States; ^4^EPHE, PSL University, Dijon, France

**Keywords:** phenotypic robustness, canalization, developmental stability, fluctuating asymmetry, shape, GWAS, epistasis, geometric morphometrics

## Abstract

The genetic architecture of skull shape has been extensively studied in mice and the results suggest a highly polygenic and additive basis. In contrast few studies have explored the genetic basis of the skull variability. Canalization and developmental stability are the two components of phenotypic robustness. They have been proposed to be emergent properties of the genetic networks underlying the development of the trait itself, but this hypothesis has been rarely tested empirically. Here we use outbred mice to investigate the genetic architecture of canalization of the skull shape by implementing a genome-wide marginal epistatic test on 3D geometric morphometric data. The same data set had been used previously to explore the genetic architecture of the skull mean shape and its developmental stability. Here, we address two questions: (1) Are changes in mean shape and changes in shape variance associated with the same genomic regions? and (2) Do canalization and developmental stability rely on the same loci and genetic architecture and do they involve the same patterns of shape variation? We found that unlike skull mean shape, among-individual shape variance and fluctuating asymmetry (FA) show a total lack of additive effects. They are both associated with complex networks of epistatic interactions involving many genes (protein-coding and regulatory elements). Remarkably, none of the genomic loci affecting mean shape contribute these networks despite their enrichment for genes involved in craniofacial variation and diseases. We also found that the patterns of shape FA and individual variation are largely similar and rely on similar multilocus epistatic genetic networks, suggesting that the processes channeling variation within and among individuals are largely common. However, the loci involved in these two networks are completely different. This in turn underlines the difference in the origin of the variation at these two levels, and points at buffering processes that may be specific to each level.

## Introduction

In recent years, studies exploring the genetic basis of skull shape have proliferated due to advances in high-throughput phenotyping techniques (Bromiley et al., 2014; Young and Maga, 2015) and genomic data collection (Flint and Eskin, 2012). The study of shape is often focused on the differences among group means, these groups being for instance species or experimental treatments. This approach has allowed the identification of a large set of genes associated with variation in mean shape in the mammalian skull (Schoenebeck and Ostrander, 2013; Weinberg et al., 2018). In contrast, only few studies have explored the association between genetic variation and shape variance (Hallgrímsson et al., 2018), likely because mapping variance is computationally more demanding [but see for example Corty and Valdar (2018) for recent methodological advance] and requires larger sample sizes than the mean. As a result, little is known about the genetic basis and architecture of shape variance.

Phenotypic robustness can be defined as the ability of an organism to buffer the impact of internal (e.g., genetic variation) and external factors (e.g., environmental effects) on the phenotype. It has been the subject of a vast literature (reviewed in Debat and David, 2001; De Coster et al., 2013; Hallgrímsson et al., 2018). Two processes have been suggested to contribute to phenotypic robustness: developmental stability and canalization (Zakharov, 1992; Debat et al., 2000; Gonzalez et al., 2016). Developmental stability, defined as the ability to buffer random developmental noise (Debat and David, 2001; Gonzalez et al., 2016), is usually assessed by fluctuating asymmetry (FA; Palmer and Strobeck, 1986). Canalization is defined as the ability to buffer the phenotypic effects of mutations (i.e., genetic canalization) and the environment (i.e., environmental canalization) [see Meiklejohn and Hartl (2002) for a discussion]. Canalization has been traditionally quantified by the variation among individuals (e.g., Hallgrímsson et al., 2018).

Whether such a partition of phenotypic robustness into these two processes is biologically justified or rather reflects a mere methodological or semantic dichotomy is an open question. The link between developmental stability and canalization has been investigated in a diversity of models using geometric morphometrics and comparing the patterns of shape variation within and among individuals. In such studies, a similarity of patterns of shape variation is typically considered indicative of a similarity of developmental processes. The results obtained have been contrasted, ranging from complete congruence to strong divergence, and no consensus on the relative status of developmental stability and canalization has been achieved [e.g., Klingenberg and McIntyre, 1998; Debat et al., 2000; Hallgrímsson et al., 2002; Willmore et al., 2006; Takahashi et al., 2010; Breno et al., 2011; see Klingenberg (2015) for a review].

A large list of candidate genes has been associated with mouse craniofacial features by either genome-wide association studies or candidate gene experiments. The highly polygenic architecture of skull shape is widely acknowledged but there is no consensus regarding whether additive effects (Pallares et al., 2016; Weinberg et al., 2018) or epistatic effects (Hallgrímsson et al., 2014) are the predominant factors influencing skull shape. Most candidate genes associated additively with shape have developmental roles (Maga et al., 2015; Pallares et al., 2015b). In contrast, studies of developmental stability (Van Dongen, 1998; Leamy et al., 2015; Varón-González and Navarro, 2018) and canalization (Percival et al., 2017) more clearly support an epistatic basis for shape variability. Still, the knowledge of the genetic architecture of canalization and developmental stability is scarce compared to the amount of data on mean shape. Identifying the differences and similarities between the genetics of developmental stability and canalization may help to clarify their relationship.

Here we jointly investigate the genetic architecture of mean shape, canalization, and developmental stability using a single dataset. This approach allows the rigorous comparison among these three aspects of shape. We use outbred mice, thousands of SNPs, and 3D morphometric analyses of the mouse skull shape to perform a genome-wide association mapping. The loci associated with differences in mean skull shape were previously published in Pallares et al. (2015b) and the loci associated with developmental stability in Varón-González and Navarro (2018). We first estimate the genetic architecture of canalization and the associated loci. Using these data, we then address two main questions: (1) is the genetic architecture of the skull mean shape different from that of canalization and developmental stability? (2) Are the phenotypic patterns and the loci involved in the regulation of canalization and developmental stability different, indicative of different biological processes?

## Materials and Methods

### Mouse Samples

We analyzed 692 Carworth Farms White outbred mice, whose main feature is a lack of population structure (Parker et al., 2014). This dataset was previously used to map the genetic architecture of skull shape variation (Pallares et al., 2015b), and both genomic and morphometric data are publicly available (Pallares et al., 2015a). The genomic data correspond to the allele dosage for a set of 79,787 genomic markers once all markers with a maximum genotype probability lower than 0.5 or a minor allele frequency lower than 2% were discarded. The phenotypic dataset is composed of 44 3D landmarks (17 pairs and 10 unique landmarks located on the mid sagittal plan) recorded on each skull. For a detailed description of the data collection process, we encourage readers to visit the original publication (Pallares et al., 2015b).

### Quantification of Skull Shape Variation and Phenotypic Robustness

Mean shape and inter-individual shape variation (canalization) were assessed using the symmetric component of shape variation: the 132 coordinates of the 44 3D landmarks were submitted to a full Procrustes superimposition with object symmetry (Dryden and Mardia, 1998; Mardia et al., 2000; Klingenberg et al., 2002) leading to symmetric variation spreading on 3 × 17 + 2 × 10 – 4 = 67 dimensions. A principal component (PC) analysis was run on the Procrustes coordinates to remove all null dimensions. This set of PCs embedded the full symmetric variation observed among individuals. Analyzing these variables was thus equivalent but computationally more efficient than working with the complete set of 132 coordinates.

**FIGURE 1:**
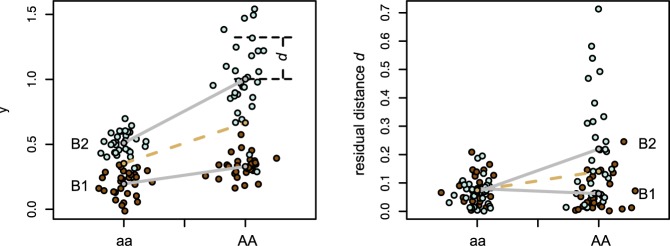
Test for genotype-specific variance. The dashed line corresponds to an additive model, the gray lines to the model integrating an epistatic interaction with the genetic background. Here, beside an epistatic interaction of the locus A with the background B on the mean phenotype, the genotype AA shows an increase in residual variance in the B2 background (left panel). This effect is captured with the epistatic modeling of the residual distance (right panel).

An individual measure of canalization was computed as the distance from each individual shape to the expected mean shape given the allele dosage at some focal SNP. To obtain this individual measure of canalization, we first estimated the effect of the focal SNP on mean shape using a multivariate linear model between the shapes and the genotypes ***y****_i_* ∼*N_q_* (***m****_i_*, ***S***) where, for a locus with an additive effect, ***m****_i_* = μ + *a_i_*α with *a_i_* is the allele dosage, α the *q*-dimensional additive effect, and **S** the residual variance–covariance matrix (Maga et al., 2015; Navarro and Maga, 2016; see Figure 1). This first step was required because we are interested in the variation around the population mean shape and many alleles associate with changes in mean (Rönnegård and Valdar, 2011). To understand why mean shape changes need to be taken into account for estimating the individual deviation, let us consider a simple hypothetical situation: imagine a given genetic marker for which one-third of the individuals are homozygous for one allele and two-third are homozygous for the alternative allele. Because of such unbalance, the population mean shape would be closer to the shape average of the majority genotype than to the shape average of the minority genotype. A simple estimation of shape variance based on distances from each individual to the population mean would thus be misleading: the individual shape distances would be systematically larger for the minority genotype than for the majority genotype, leading to an artifactual linear association between the marker and the shape variance. The effect of gene dosage upon mean shape should thus be accounted for in any estimation of shape variance.

Once the effect of the focal marker on the mean shape was inferred, we estimated the distance from each individual shape to the predicted genetic effect for mean shape as: $d_{i}=\sqrt{{\left(y_{i}-m_{i}\right)}^{t}D^{-1}(y_{i}-{\text{m}}_{i})}.$ The shape variance was thus assessed as the dispersion of the individual shapes around the predicted mean shape for their genotype. Taking ***D*** equals to identity matrix ***I****_q_* leads to the Euclidean distance. The use of distances assumes that the potential QTLs affect the *amount* of population variation but not the *direction* of that variation. To alleviate the potential implications of ignoring the direction of variation, we estimated Mahalanobis distances taking ***D*** equals to the residual variance covariance matrix ***S*** with elements $s_{jk}={\left(n-1\right)}^{-1}\sum_{i=1}^{n}{\left(y_{ij}-m_{ij}\right)}^{t}(y_{ik}-m_{ik})$ (Klingenberg and Monteiro, 2005). This distance allows accounting for the anisotropic variation typical of mammalian skull (Klingenberg, 2013), weighting more heavily the deviations that are in the directions of least variation at the population level.

### Genome-Wide Association Scans

Although the relatedness among individuals is standardized in the mapping population used here (Pallares et al., 2015b), we applied a linear-mixed model in order to control for population structure (Speed and Balding, 2015). We estimated a genomic relatedness matrix with a leave-one-chromosome-out approach, a method designed to reduce the loss of power associated with the inclusion of SNPs in both the relatedness matrix and the linear model (Listgarden et al., 2012; Cheng et al., 2013). This matrix of genomic relatedness was then used to estimate the variance components and a phenotypic covariance matrix, which in turn helped to correct both genotypic (allele dosages) and phenotypic data for each chromosome. Corrected genotypic and residual distance data were then fitted in a linear model, which now had uncorrelated errors among individuals (Nicod et al., 2016).

#### Mean Shape

Genome-wide association mapping were run on the 67 PC from the symmetric shape to estimate which loci contribute additively to the mean shape variation. Such an association should be assessed preferentially using a multivariate model because the effect of loci generally does not align with the PCs (Maga et al., 2015) and loci with a large multivariate effect may only weakly participate to all PCs because of the projection. Such an approach can be quite challenging because of the large number of covariances to be estimated. Despite the potential large drop in power, genome scans with univariate mixed model on individual PCs found numerous genomic markers additively associated to the skull mean shape (Pallares et al., 2015b). In the following, we used these published results as an estimate of the genetic basis for mean shape variation. We should nevertheless keep in mind that additional loci may also contribute in a sizeable part to the mean shape variation and that epistasis was not specifically searched for in that study.

#### Canalization

The association tests estimated whether there are loci *l* associated with the average distance from each individual to the population mean shape given the locus genotypes ${\bar{m}}_{l}=n^{-1}\sum_{i=1}^{n}a_{il}\alpha_{il}$ (i.e., whether individuals that are on average further away from the population mean ${\bar{m}}_{l}$ carry specific alleles and *vice versa*): $d_{i}\sim N(\bar{d}+a_{il}\gamma_{l},\sigma^{2})$, where γ_l_ is the additive effect of the locus *l* on the distance (Figure 1). This model looks for loci affecting environmental variance and therefore contributing to environmental canalization (Wagner et al., 1997).

#### Developmental Stability

The individual estimations of canalization used here are equivalent to the individual measures of FA calculated in a previous study (Varón-González and Navarro, 2018), where the genetic architecture of developmental stability was estimated from the asymmetric component of shape variation.

We defined two significance thresholds (5% and 10%) from the distribution of maximum negative log_10_ *p* derived from 1000 permutations of the corrected residual distances (Churchill and Doerge, 1994). Analyses were run in R version 3.4 (R Core Team, 2013).

### Marginal Epistasis Scan for Canalization

The marginal epistasis test^1^ (Crawford et al., 2017) was applied to the distance data to test for epistatic effects among genomic regions in association with shape variance. The advantage of the marginal epistasis test is that it first fits a linear model for each genomic marker with a variance component representing epistatic variance of that marker with all the rest of the markers and then only the best-fitting markers are tested in pairwise comparisons. This first selection circumvents the intractable number of pairwise comparisons for all the markers. In our case, markers with a *p* < 10^-6^ in the Davies method were selected for further pairwise evaluation. The marginal epistasis tests were computed on Mahalanobis distances from the overall mean shape (i.e., ***m****_i_* = μ in the equations above) because they look simultaneously at a large number of genotypes. In contrast, and similarly to the additive genome scan, pairwise interactions were corrected for the effect on the multivariate genotype means and then ran on residual Mahalanobis distances. The interactions were considered significant when *p* < 0.001. Because our data do not show any significant effect of relatedness among individuals and the epistasis test is relatively robust against it (Crawford et al., 2017), no correction for population structure was applied. Only first-order epistasis was considered for two practical reasons. First, the inclusion of higher-order interactions would exponentially increase the number of potential interactions and their complexity. Second, these estimations would be increasingly data-hungry. Our sample comprises many individuals with one specific combination of just two markers but comparatively few individuals sharing specific combinations of many markers. This sample reduction for certain estimations would increase the relative importance of inaccuracies in our measures of developmental stability or canalization, affecting the reliability of the results.

### Investigating Gene Ontology and Representing Genetic Networks

Our sample presents less linkage disequilibrium than other populations of mice (Nicod et al., 2016). Therefore, for a given marker we defined its associated candidate genes as all the genes closer to it than 200 kb. This window size would correspond to a correlation among markers (i.e., linkage disequilibrium) of about 0.6. For each marker found in a significant interaction we collected all of its overlapping candidate genes annotated in the *Mus musculus* reference genome (version m38.92) in Mouse Genome Informatics (Blake et al., 2017) and Ensemble (Yates et al., 2016). When no protein-coding genes were found, we reported other genomic elements, i.e., RNA genes, processed transcripts, and pseudogenes. Markers overlapping the same list of genes were merged in a single genomic region.

We obtained two reference gene lists in the MGI HMDC database searching for “Craniofacial” and “Growth/Size/Body” (http://www.informatics.jax.org/humanDisease.shtml; accessed 4 April 2018). Once redundancies between lists and human-specific genes were removed, we were left with 1044 and 2829 genes for “Craniofacial” and “Growth/Size/Body” categories, respectively. Then, we assessed the over-representation of candidate genes within the two lists with Fisher’s exact tests (Fisher, 1935).

Based on the results from the marginal epistasis test, we built a network to reflect the significant pairwise interactions among genomic markers associated with shape variance. To illustrate the role of each candidate gene we classified them as “Craniofacial” or “Growth/Size/Body” depending on the reference gene list in which they appear. The rest of protein-coding genes were labeled as “Other” and regulatory candidate genes as “Regulatory.” Hub scores, power, and betweenness centralities of the genomic regions in the epistatic network were computed to check for candidate genes with important roles within the networks and therefore to look for major epistatic controllers. Kleinberg’s hub centrality scores are the principal eigenvector of ***A*** ×*t*(***A***), where **A** is the adjacency matrix (Kleinberg, 1999). It is therefore a measure of the node’s connectivity. The betweenness centrality of a node, estimated as the number of times the shortest path between all possible pairs passes through that node, is correlated to the hub score, as the more connected one node is (hub score) the more paths will pass through it (betweenness). Finally, the power centrality for each candidate gene is estimated with the Bonacich’s (1987) approach: the algorithm computes the importance of a given node as a function of the number of its first and second-degree connections. A hub will be considered more important if it has many adjacent connections that are relatively isolated than if it is just another node within a complex and highly connected network. Positive values represent a cooperative relation with its connections, so the hub would be more powerful as its connections would also become more powerful; negative values are typical of antagonistic relationships. These measures were computed using the igraph package^2^ (Csardi and Nepusz, 2006).

### Estimating and Comparing the Patterns of Shape Variation

Based on the same mouse population and phenotypic and genomic data used here, previous studies have mapped the loci contributing to skull mean shape (Pallares et al., 2015b) and those contributing to skull shape FA (Varón-González and Navarro, 2018). Given that the three studies use not only the same mouse sample, but also the same genomic markers and overall approach to deal with population structure, the comparison between the results is unbiased and straightforward.

### Estimating G and E Matrices

The additive genetic covariance matrix (**G** matrix) corresponds roughly to the crossproduct of the frequency-weighted additive effects of all causal loci (Kelly, 2009). **G** was estimated from the genomic relatedness matrix using a linear mixed model on the symmetric component of shape variation and implemented in the sommer R package (Covarrubias-Pazaran, 2016). The patterns of shape variation described by **G** result from the interplay of additive genetic variance and the buffering effects of genetic canalization. The linear mixed model also provided the residual variance-covariance matrix **E** which results from the interplay between the non-additive genetic variance (part of the genetic variance not accounted for in the estimation of **G**), environmental variance, and the buffering effects of genetic and environmental canalization.

Because of the large dimensionality of the shape data, we used a block strategy to process first PC1 to PC10 (accounting for 62.3% of the total variance), which were estimated simultaneously together with their correlations, and then a second block from PC11 to PC67 (in average 0.66% of the total variance), which was modeled with diagonal variance components (i.e., with covariances set to zero) and allows to fit independently these 51 additional PCs. This strategy represents a compromise between an ideal but intractable solution estimating every dimension simultaneously and an efficient but formally incorrect approach assuming a complete match between genetic and phenotypic PCs, an assumption that is, in most cases, unrealistic. Another option would have been factor analysis (Meyer, 2009; Runcie and Mukherjee, 2013). This approach does not enforce any genetic correlations between phenotypic directions to zero, but rather forces the genetic variation to spread on a limited number of traits. While this could be relevant for some phenotypes, it may not be appropriate to inherently multivariate shapes, which show heritable variation in almost all directions (e.g., Klingenberg et al., 2010; Navarro and Maga, 2016).

### Estimating P and FA Matrices

We investigated the patterns of shape variation at two more levels: the global phenotypic variation present in the sample as the phenotypic covariance matrix **P** and the stochastic component of shape variation with the covariance matrix of shape FA. **P** and **FA** covariance matrices were estimated from the superimposed Procrustes coordinates of the symmetric and asymmetric components of skull shape, respectively (Klingenberg et al., 2002). **P** results from the interplay between genetic and environmental influences on the phenotype and the buffering effect of canalization (i.e., genetic and environmental canalization). Similarly, the covariance matrix of shape FA results from the interplay between stochastic developmental noise affecting the two sides independently, and the buffering effect of developmental stability.

The heritability of shape was assessed with the general multivariate heritability formula: ***h*^2^** = ***GP*^-1^** (Lande, 1979). The main directions (i.e., eigenvectors) of the **GP**^-1^ matrix display shape features with the highest heritability. Therefore, these eigenvectors represent the shape changes that can potentially respond more rapidly to selection when the additive variance is high (Klingenberg and Leamy, 2001). These axes have been sometimes referred to as lines of evolutionary least resistance in the literature (Schluter, 1996), although this may not be relevant in absence of additive effects (Uller et al., 2018) as gene interactions should be taken into account to predict selective responses.

### Matrix Comparisons

As **G** and **P** mostly differ by the environmental component of variation, included in **P** but not **G**, comparing them can tell us whether the environmental effects on shape variation fundamentally differ from those of genetic differences. Besides, comparing **G** and **P** is of interest in the context of evolutionary studies of selection: it has been suggested that **P** could be used as a surrogate for **G**, in cases where the estimation of **G** is not tractable (in non-model species for example). The validity of such an approach depends on the similarity between **G** and **P**, often assumed but seldom assessed (Bégin and Roff, 2004).

Comparing **P** and **FA** matrices has been used as an indirect test of the relationship between canalization and developmental stability: similar patterns of shape variation would be indicative of a similarity in the processes generating them (e.g., Klingenberg and McIntyre, 1998; Debat et al., 2000; Breuker et al., 2006). Canalization appears nonetheless more adequately measured after controlling for genetic and environmental variation (Hallgrímsson et al., 2018). Environmental variation is not controlled in our design besides randomization across individuals, and non-additive variance components are not estimated but in any case **E** appears more adequate than **P** to measure and compare canalization to developmental stability. **E** precisely describes the patterns of the shape variation that is used in the additive genome scan but it differs from the residual variation used in the epistasis tests where this non-additive variation was modeled.

There are many methods and metrics, more or less redundant, that allow comparing matrices (e.g., Teplitsky et al., 2014; Roff et al., 2012). A global distinction can be made between (1) methods deriving a single measure of global similarity (e.g., distances, correlations), which may or may not be sensitive to scaling and orientation, (2) methods based on the individual spectral decomposition of each matrix (e.g., comparison of the first eigenvectors), which can be very sensitive to the orientation, and (3) methods based on a common spectral decomposition (e.g., Krzanowski’s subspace analysis), which capture the overall similarity together with a decomposition of the matrix divergences. Subspace analyses [Krzanowski (1979); see Aguirre et al. (2014) for a recent application to **G** matrices] are much more informative than single metrics and are not biased by differences in orientation, contrary to comparisons based on individual spectral decomposition, which might be misleading because of trivial rank-order permutations or rotations of axes. Covariation patterns can indeed be globally similar despite the occasional lack of a strict match between PCs.

To assess similarities among patterns of covariation, we first computed the Krzanowski subspace of the set of the four matrices **M***_i_*:{**P**, **G**, **E**, **FA}** matrices. In addition to the overall simplicity of the estimations, this method uses the data contained in the covariance matrices and does not require further assumptions. The common subspace among the four covariance matrices is found with a simple equation: $\text{H}=\sum_{i=1}^{p}{\text{A}}_{i}{\text{A}}_{i}^{t},$ where **A***_i_* is a matrix with a subset of the eigenvectors of **M***_i_* as columns. For the eigenvalues Δ of **H** less than *p* (here *p* = 4, the number of matrices to compare), at least one population cannot be inferred from a linear combination of the eigenvectors of **M***_i_* defining the subspace. We can estimate how similar the corresponding eigenvector ***b****_k_* of **H** is to each population’s subspace with the angle ${\text{δ}}_{i}=\cos^{-1}\{{\left(b_{k}^{t}A_{i}A_{i}^{t}b_{k}\right)}^{0.5}\}$. This estimation was obtained using the evolqg R package (Melo et al., 2016).

For comparison with earlier studies (e.g., Debat et al., 2000), we also computed overall similarity measures based on distances, correlations, and differences in integration. We then checked for a strict similarity of the main axis of variation computing the angles among the first eigenvectors. The overall distance among the four covariance matrices was measured as their pairwise Euclidean distance $d_{E}(S_{i},S_{j})=||S_{i}-S_{j}||=\sqrt{\text{trace}\{{\left(S_{i}-S_{j}\right)}^{t}(S_{i}-S_{j})\}},$ where ***S****_i_* is the sample covariance, symmetric, and positive semi-definite (Dryden et al., 2009). This distance is one of the possible measures applied in morphometrics (Klingenberg, 2013). We also computed matrix correlations based on element-wise correlations (Klingenberg and McIntyre, 1998). Both Euclidean distances and matrix correlations were based on the upper-triangular parts of the matrices only (including the block diagonal) because the off-diagonal elements would have appeared twice otherwise (Klingenberg and McIntyre, 1998). Significance of matrix correlation was tested by 10,000 random permutations of the landmarks. Triplets of *xyz*-coordinates were permuted together to preserve their association at each landmark.

The structure of the four matrices was further compared based on their degree of integration. Integration describes the strength of covariation among shape variables: the stronger the integration, the higher the variance that can be explained by a single linear combination of the shape variables (i.e., the first eigenvector). The strength of integration can thus be estimated by the unequal distribution of variance across dimensions of the shape space (i.e., decrease of eigenvalues; Wagner, 1984). We assessed integration accordingly as the ratio of the first two eigenvalues (referred to as the eccentricity, Jones et al., 2003).

Finally, we assessed the similarity of the patterns of maximal variation by estimating the angles among the first eigenvectors. Significance testing was achieved by comparing these angles to a set of 100,000 random angles of the same dimensionality (Klingenberg and McIntyre, 1998).

Shape spaces for FA and symmetric shapes are in orthogonal subspaces (Klingenberg et al., 2002) and unpaired landmarks were therefore discarded before comparisons. Comparisons between matrices derived from the symmetric shapes were also based on half configuration: one half of the paired configuration plus the unpair landmarks, as the paired landmarks would have appeared twice otherwise.

## Results

### Canalization Is Associated With an Epistatic Network

**FIGURE 2:**
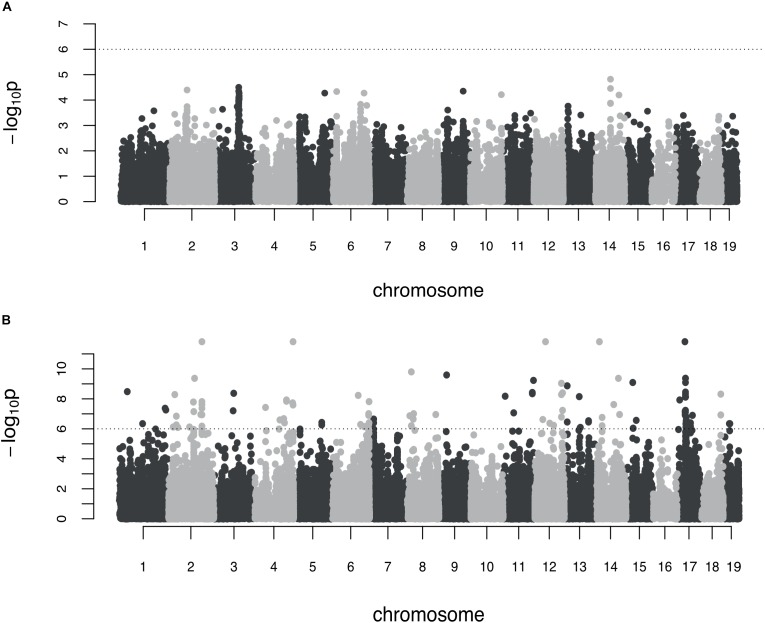
Genome-wide association on the symmetric shape variance (environmental canalization). **(A)** Additive genome scan. Each dot corresponds to the LOD score of one marker and the dashed line represents the significance threshold; **(B)** marginal epistasis genome scan (Davies method). The dashed line corresponds to the threshold set to select marker for subsequent pairwise analyses.

No association was found between genetic markers and shape variance when the additive genetic scan was used (Figure 2). In contrast, the epistasis test identified a preliminary set of 118 genomic markers (*p* < 10^-6^) to be explored further in pairwise analyses. The genetic architecture of skull shape canalization thus appears purely epistatic.

**Table 1:** Top-associated SNP in marginal epistasis genome scan (*p* < 10^-9^).

SNP	Chr	Pos (Mb)	*P*	Candidate genes	Role
cfw-2-105373402	2	105.37	4.23 × 10^-10^	Wt1	Growth/size/body
rs27267349	2	134.53	<10^-10^	Hao1/Tmx4	Other
rs220408352	4	153.84	<10^-10^	A430005L14Rik/Dffb/Cep104/Lrrc47/Smim1/Ccdc27	Other
rs108441213	8	19.98	1.58 × 10^-10^	Gm31371	Other
cfw-9-31091829	9	31.09	2.48 × 10^-10^	St14	Craniofacial
cfw-11-118979964	11	118.98	5.89 × 10^-10^	Cbx2	Craniofacial
rs257536585	12	44.95	<10^-10^	Stxbp6	Other
rs224184036	12	108.10	9.23 × 10^-10^	Setd3	Growth/size/body
rs6258161	14	18.39	<10^-10^	Nr1d2/Rpl15/Nkiras1/Ube2e1/Ube2e2	Other
rs32308587	14	93.62	4.25 × 10^-10^	Pcdh9	Other
rs32065990	15	26.22	8.22 × 10^-10^	March11	Other
cfw-17-30673769	17	30.67	<10^-10^	Btbd9	Growth/size/body
cfw-17-32460913	17	32.46	8.02 × 10^-10^	Brd4	Craniofacial
rs29531435	17	32.94	4.37 × 10^-10^	Cyp4f14	Growth/size/body
rs216241249	17	32.96	4.20 × 10^-10^	Cyp4f14	Growth/size/body

From the 15 markers that best associate with the general epistatic variance (*p* < 10^-9^), three overlapped with genes known to contribute to craniofacial variation, five with genes having an effect on growth, and seven with other protein-coding genes (Table 1). Although only two of the markers (rs216241249 and rs29531435) seemed to overlap regarding their best candidate gene, these 15 best-associated markers did not appear equally spread across the genome (four markers in chromosome 17 and two in chromosomes 2, 12, and 14).

In the second step of the epistasis test, where specific pairwise interactions were associated with shape symmetric variance, 385 pairwise interactions involving 111 genomic markers (80 when the markers with equal sets of candidate lists were merged) showed significant associations. These significant genomic markers overlapped with 893 candidate genes, 413 of which were protein-coding genes. Fisher’s exact test showed a significant over-representation of craniofacial genes (*p* = 2.1 × 10^-6^) as well as growth genes (*p* = 3.07 × 10^-4^) among these significant interactions. For example, one marker detected in the three most significant pairwise interactions was found overlapping with *Rapgef2*, a gene involved in abnormal size and neural features (Wei et al., 2007). From the significant 111 markers, 30 were classified as “Craniofacial,” 33 as “Growth,” 43 as “Other,” and 5 as “Regulatory.” These proportions were similar even after merging regions that overlapped: out of 80 markers 16 were classified as “Craniofacial,” 23 as “Growth,” 36 as “Other,” and 5 as “Regulatory” (χ^2^ = 1.68, *p* = 0.64).

**FIGURE 3:**
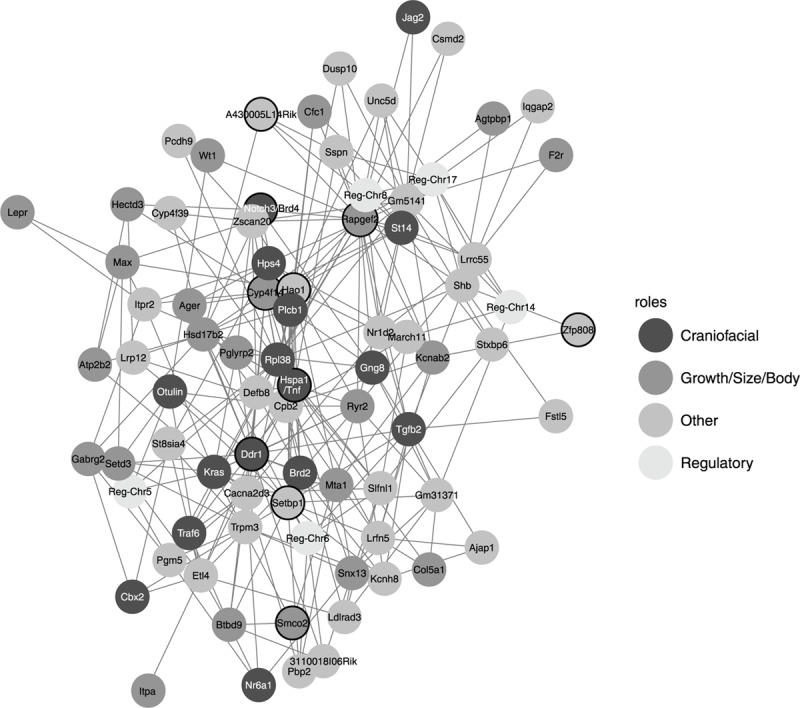
Gene network based on the significant pairwise interactions obtained with the marginal epistasis test. Each node corresponds to the best candidate gene of one marker and its color represents the function of that candidate gene: candidate genes found in MGI HMDC databases for “Craniofacial” and “Growth/size/body” functions are classified in these two groups, the rest of protein-coding genes are labeled as “Other” and those genes that are not protein-coding are classified as “Regulatory.” Black circles correspond to candidate genes with high centrality measures and are discussed in the main text.

Among the genomic markers appearing in significant pairwise interactions, 10 appeared in more than 16 interactions (Figure 3). Two of these highly connected markers overlapped with genes involved in craniofacial features, *Tnf* (20 interactions) (Alayan et al., 2007) and *Ddr1* (17 interactions) (Dullin et al., 2007). The *Tnf* gene is indeed near *Hsp1a*, which encodes for the chaperon protein Hsp70: a protein that has been suggested to play a role in canalization in *Drosophila* (Takahashi et al., 2010). Two other of these markers were also overlapping growth genes as *Rapgef2* and *Cyp4f14* (Blake et al., 2017). The latter is the most connected candidate gene when we merged the genomic markers with the same candidate gene lists. Within this reduced list of genomic markers we also found *Tnf* and *Hao1*/*Tmx4* (Visel et al., 2004; Masuda et al., 2009), as well as other new candidate genes as *Brd4* (Houzelstein et al., 2002), *Kras* (Hernandez-Porras et al., 2014), or *Plcb1* (Ballester et al., 2004).

**FIGURE 4:**
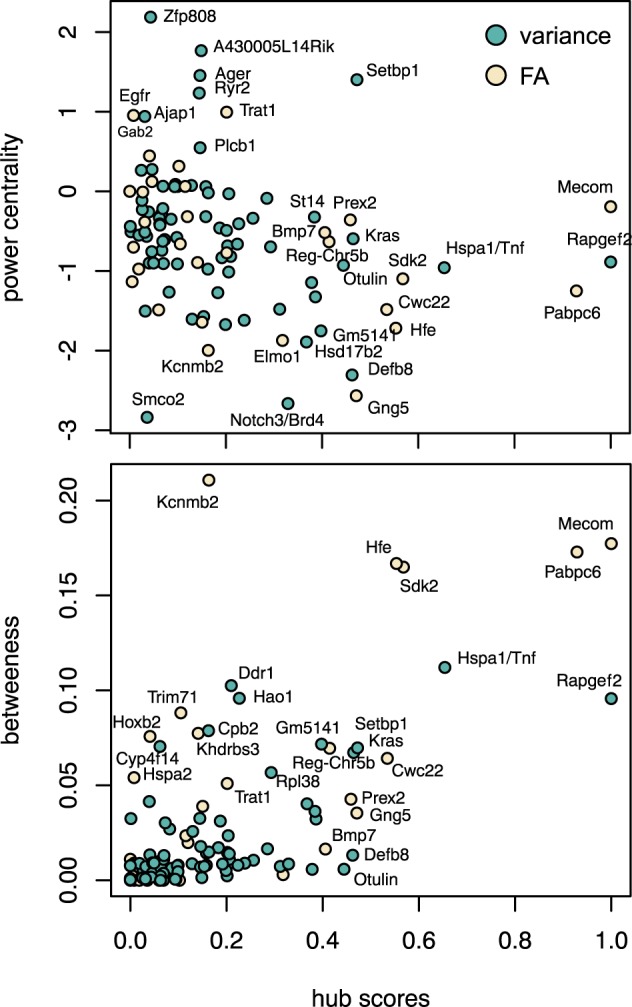
Centrality of interactive gene candidates in developmental stability and the canalization networks based on hub scores, power centrality, and betweenness.

The analysis of the network obtained from the pairwise interactions showed different features among the epistatic candidate genes (Figure 4). *Rapgef2* and *Hsp1a* had the largest hub scores, so they showed the largest connectivity within the network. Because hub scores and betweenness centrality are correlated, these two candidate genes also had very high betweenness centrality, although *Hsp1a* showed the lowest hub score and the highest betweenness centrality among the two. *Ddr1* and *Hao1* also showed very high centrality although relatively low hub scores: these genes might not interact much but they might interact with other genes that do interact a lot and therefore both *Ddr1* and *Hao1* may regulate important hubs. The power centrality is different, and candidate genes as *Zfp808* and *A430005L14Rik* (on the positive side) or *Smco2* (Blake et al., 2017) and *Notch3/Brd4* (on the negative side) (Karst et al., 2010) showed high power centrality but low hub scores. We should note also *Setbp1* (Piazza et al., 2018) which presented high power centrality and average hub score.

### The Genomic Markers for Mean Shape, Canalization, and Developmental Stability Are Different

In a previous study, Pallares et al. (2015a) detected 17 loci additively associated with the skull mean shape that explained about 45% of the total phenotypic variance. For developmental stability, Varón-González and Navarro (2018) did not detect any additive effect but rather a large number of loci epistatically associated with FA. From these two studies, it appears that the mean shape and its developmental stability do not share the same genetic architecture nor the same loci (Varón-González and Navarro, 2018).

None of the significant epistatic markers associated with canalization were common to mean shape or developmental stability. Indeed, when instead of the genomic markers we compared the genomic regions associated with each significantly associated marker (see the section “Materials and Methods,” Investigating Gene Ontology and Representing Genetic Networks), we only obtained 11 common protein-coding candidate genes between canalization and developmental stability: *Arhgef4* (Blake et al., 2017), *Chd5* (Diez-Roux et al., 2011), *Kcnab2* (Perkowski and Murphy, 2011), *Nphp4* (Won et al., 2011), *Plekhg3* (Diez-Roux et al., 2011), *Sptb* (Bernstein, 1980), *Churc1* (Diez-Roux et al., 2011), *Fntb* (Blake et al., 2017), *Rab15* (Blake et al., 2017), *Max* (*Hspa2*) (Shen-Li et al., 2000), and *Gpx2* (Diez-Roux et al., 2011).

Despite this overlap, the structure of the epistatic networks was very different: the common protein-coding genes did not show similar hub scores and connectivity (Figure 4). For FA, the candidate genes *Mecom* (Hoyt et al., 1997) and *Pabpc6* (Diez-Roux et al., 2011) have the highest hub scores and very high betweenness centrality, equivalently to *Rapgef2* and *Hsp1a* for canalization. We should note that *Mecom* and *Setbp1*, which have high power centrality in the canalization network, are thought to interact in a positive feedback loop (Piazza et al., 2018). The candidate genes that showed the highest and lowest power centrality within the FA network, *Egfr* (Miettinen et al., 1999), *Gab2* (Wada et al., 2005), and *Trat1* (Kolsch et al., 2006) on the positive side and *Gng5* (Moon et al., 2014), *Kcnmb2* (Martínez-Espinosa et al., 2014), and *Elmo1* (Blake et al., 2017) on the negative side, were not shared by the canalization network either.

### Genetic, Phenotypic, and FA Covariance Matrices Are Mostly Similar

**FIGURE 5:**
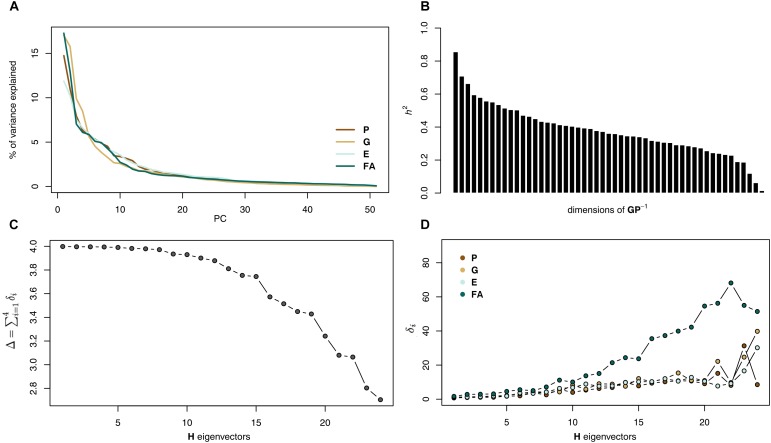
Heritability and covariance matrices analyses. **(A)** Heritability results: amount of heritability for each dimension. **(B)** Amount of variation for each dimension in the G, FA, and P covariance matrices. **(C)** Results from the Krzanowski analysis: variation of *D* (left) and of the angles *δ* (right) with the inclusion of eigenvectors. **(D)** Two-dimensional representation of the **G**, **FA**, and **P** covariance matrices. G shows less eccentricity (more integration) than the FA and P matrices and G and P shows a more similar pattern of variation (vertical in this example) than any of them with FA.

The analysis of the **GP^-^**^1^ matrix showed that heritability is spread across many directions of the shape space (Figure 5b). Most of these directions (91%) showed heritability higher than 0.2 and a third of them (21 dimensions) accounted for more than 1% of the total additive variance. This means that these directions are actually operational for selection since they present substantial additive genetic variance.

The main patterns of variation appeared very similar among all matrices: the Kraznowski subspace analysis returned almost perfect *Δ* values for about half of the subspace (*Δ*_1-5_ > 3.99 and *D*_1-11_ > 3.90, which must be compared to a maximum value of 4; Figure 5c). For these dimensions, the angles *δ* between the subspace eigenvectors and the individual matrices were very small (*δ*_1-5_ < 5° and *δ*_1-11_ < 14°) showing the very good agreement between the variation described in the subspace and each reduced matrix (Figure 5d). The *D* value dropped afterward due to the divergence of the **FA** matrix, its *δ* angles deviating more, until reaching a value of 3 at dimension 22 over the 24 possible dimensions. When **FA** was excluded, the maximum *D* value (here 3) was obtained for all dimensionalities. These results suggest very similar patterns of variation among the first matrices eigenvectors, but then a marked difference of **FA** when the dimensionality becomes larger.

Analyses based on overall similarities between matrices suggested that FA is quite different (Table 2). Both the Euclidean distance and the element-wise matrix correlation supported this. Including the unpaired landmarks did not affect the comparison of the **PGE** matrices. **P** and **FA** matrices showed similar eccentricity (**P** = 1.80 and **FA** = 1.78), while the **G** matrix showed a more spherical shape (1.17) and **E** was intermediate (1.34). The greater sphericity of **G** is apparent beyond the two first PCs with a slower decreasing of the proportion of variance explained by each PC for this matrix than for **P**, **E**, and **FA** (Figure 5a). When the midline landmarks were added, **G** appeared more similar to **P** (2.85 and 2.69, respectively) and more concentrated than **E** (2.18) and **FA** was even more concentrated (3.76).

The comparison of the angles between the first eigenvectors yielded striking differences: **P**, **G**, and **E** eigenvectors were more similar than random vectors (Table 2) but this was not the case with the **FA** vector, which was no more similar to **P**, **G**, and **E** vectors than to random vectors. This result was partly due to a rank-order permutation between the first PCs as the angles between **FA** PC1 and PC2 from **P**, **G**, and **E** (74.2, 55.8, and 67.5°, respectively) are smaller than those observed between the PC1s, and the vectors are significantly more related to each other than two random vectors. The matches are nonetheless not perfect and it thus appears more adequate to consider several PCs altogether to get a less biased comparison of the patterns of shape variation.

**FIGURE 6:**
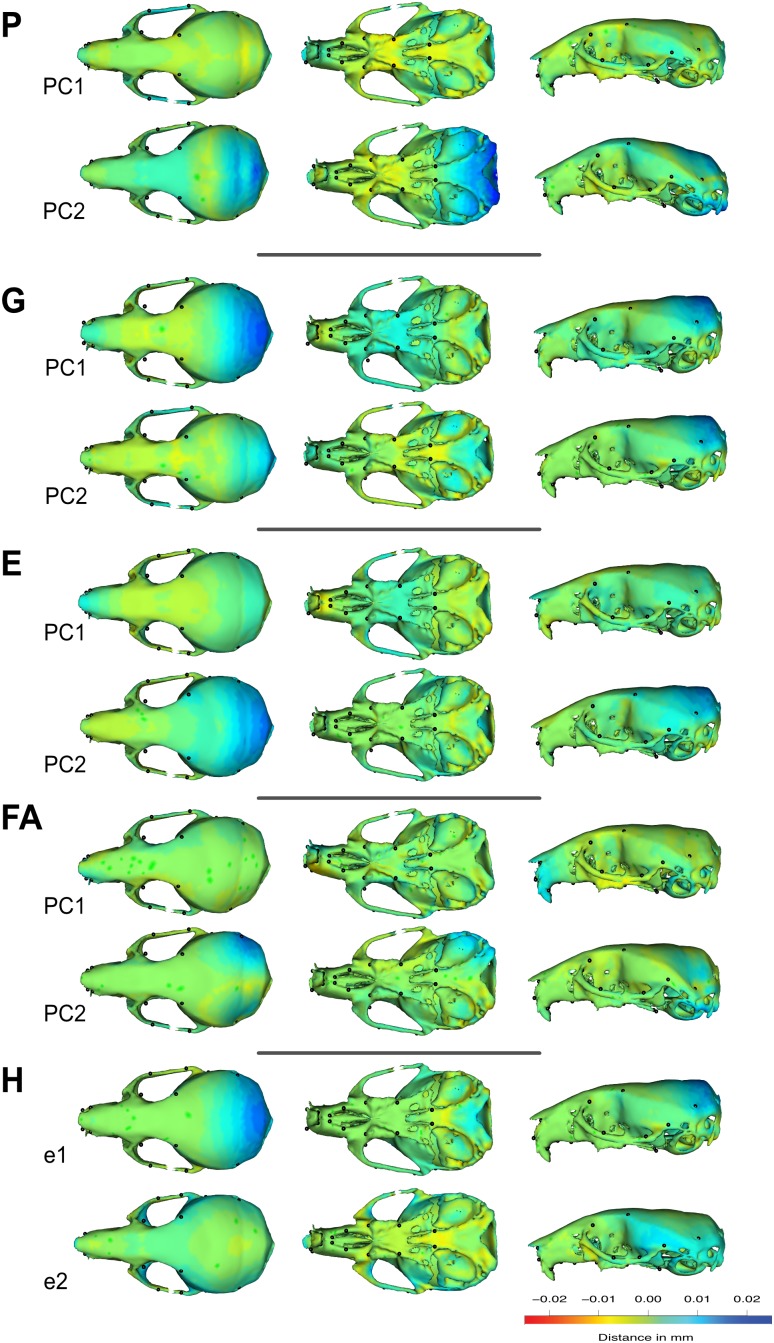
Visualization of the shape changes associated to the first eigenvector of the **G**, **FA**, and **P** matrices. Here, we present variation in three perspectives of the same skull. Color differences represent the areas and intensity of the variation expressed by the first or second eigenvector of the matrices (green: no variation; blue: expansion compared to the mean shape; red: contraction compared to the mean shape). As symmetric and asymmetric spaces are orthogonal, the estimation of the first eigenvector excluded the unpaired landmarks located on the mid plane of the skull. Comparison between FA patterns and others should be done on only one side of the skull.

Krzanowski subspace analysis should be favored over other methods: by estimating shared linear combinations it handles many of the possible trivial transformations that could arise between matrices and bias the comparison. Overall, we thus report a very strong congruence between the patterns of shape variation as described by the different matrices, and in particular between individual variation and FA within a large subspace, despite the limited similarity across major axes (PCs). The congruent pattern of shape changes shared across these axes (i.e., the eigenvectors of the subspace **H**) corresponds mainly to an expansion of the posterior part of the skull (Figure 6).

## Discussion

Skull shape is a polygenic multivariate trait associated with a vast number of genes and regulatory elements contributing to craniofacial and growth developmental pathways. Despite the large amounts of non-linear dynamics and epistasis in these pathways (Hallgrímsson et al., 2014) loci acting additively were detected (Pallares et al., 2015b). In this study, we found that developmental stability and canalization of skull shape are also influenced by a large number of genes, a set significantly enriched for protein coding genes known to contribute to craniofacial diseases, but contrary to mean shape, they present an entirely epistatic genetic architecture. The genetic networks underlying the two components of phenotypic robustness are strikingly different, as they share very few candidate genes. Developmental stability and canalization however showed very similar patterns of phenotypic variation.

### Methodological Limits: Matrix Comparisons and Higher Order Epistasis

#### Matrix Comparisons

It is conceivable that part of the incongruence among studies investigating the relationship between canalization and DS might be related to the fact that similarities among patterns of variation are sometimes inferred strictly on the first few PCs (e.g., Klingenberg and McIntyre, 1998; Debat et al., 2000). The present study shows that the main pattern of covariance might be similar, with a nevertheless marked divergence between the PCs (e.g., **FA** PC1 is clearly different, but the global subspace is very similar). This is simply due to a global rotation of the axes: considered together, they contain roughly similar information, but they individually depict different aspects of shape variation (i.e., the variation is differently spread over the PCs). Although such rotation nevertheless indicates a phenotypic difference, it can completely mask the overall similarity. On the other hand, distances or correlation and subsequent multivariate ordination of matrices based on such metrics (e.g., Debat et al., 2006; Mitteroecker and Bookstein, 2009) may also be misleading because of their over-integrative nature.

**Table 2:** Similarities between matrices.

		Half-configuration^1^				Half-config. + unpaired landmarks				
	*d*_E_ (×10^-5^)	*r*	*p*_r_	α (°)	*p*_α_	*d*_E_ (×10^-5^)	*r*	*p*_r_	α (°)	*p*_α_
P–G	1.69	0.95	<0.0001	53.93	<0.0001	3.32	0.96	<0.0001	14.51	<0.0001
P–E	1.30	0.93	<0.0001	39.05	<0.0001	2.60	0.91	<0.0001	46.24	<0.0001
G–E	0.72	0.80	<0.0001	66.01	0.003	1.53	0.80	<0.0001	37.48	<0.0001
P–FA	5.30	0.64	<0.0001	81.21	0.28					
G–FA	1.85	0.60	<0.0001	89.13	0.92					
E–FA	1.91	0.67	<0.0001	83.99	0.46					

*^*1*^Similarities between matrices are computed on half configuration of the paired landmarks with or without the unpaired landmarks on the mid plan of the skull. Variation at these unpaired landmarks is orthogonal between symmetric and asymmetric shapes and their comparison is meaningless*.

Comparisons allowing both the assessment of the overall similarity and a hierarchical evaluation of this similarity such as the Krzanowski subspace should systematically be performed in conjunction with individual PCs or distance comparisons. In the present study, the subspace analysis indicated very strong similarities among all matrices for the main dimensions, although **FA** was more divergent at higher dimensions. This divergence could be genuine but could also result from the greater uncertainties in FA estimation compared to among-individual variation. Results from matrix distances or correlations depict a similar picture of relative similarities but miss the large common ground for the main patterns of variation. Emphasizing the importance of such a slight divergence would lead to consider canalization and developmental stability as distinct. On the contrary, the overall similarity observed among the main patterns of **FA** and phenotypic covariances suggests that variation stemming from different sources is similarly channeled throughout development along the same lines of shape variation in adults. This results plead for a similarity in the channeling processes (see below).

#### Higher Order Epistasis

Could the discrepancy between mean, canalization and development stability genetic networks be due to the fact that only first-order interactions were considered? This might indeed limit the resolution of the inferred networks: using higher order interactions might potentially allow detecting many additional genes and genetic interactions with connections reflecting smaller effects. As explained in the recent omnigenic theory (Boyle et al., 2017), pushed at its limit, this approach would ultimately identify networks including most or even all the genes of the genome, as all the genes are in a way or in another related to the development of complex traits. In that case, the direct comparison of the node networks might not be very informative and sensible information may rather be searched in wiring motifs for example. In practice, given the large differences in the genome scans we have found between the mean shape and the components of shape robustness, we would not expect higher-order interactions to change our main results that large additive components are found for shape but not for shape robustness.

### Mean Shape and Shape Variability Have Different Genetics Bases

The genetic architecture of skull shape has been extensively studied. Although epistatic effects have been occasionally reported when searched [e.g., Wolf et al., 2005, 2006; see Hallgrímsson et al. (2014) for a discussion], additive effects have been frequently found and frequently proposed to play a significant role in shape (Pallares et al., 2014, 2015b; Maga et al., 2015; Weinberg et al., 2018). In contrast, this study and previous studies on FA (e.g., Leamy and Klingenberg, 2005; Burgio et al., 2009) point at a purely epistatic genetic basis for both FA and individual variation, suggesting a different genetic basis for the skull mean shape and its variability.

Additive and epistatic variations are not mutually exclusive as they refer to two statistical models and some genes can show additive effects when they have a strong effect in their interactions (Mackay, 2014; Huang and Mackay, 2016). Inversely, purely additive effect on individual variance or FA could be too small to be detected with our experimental design. In addition, the complexity of developmental processes can mask additive signals if for example one molecule expressed later in development overwrites a previous phenotypic signal, a likely situation given the non-linear properties of development (Hallgrímsson et al., 2009; Green et al., 2017). Finally, additive effects have been found for phenotypic robustness in QTL experiments (Gonzalez et al., 2016). The genetic architecture for mean shape and shape variance might thus be more similar than suggested by our results.

The contrast between the strong additive effects found in Pallares et al. (2015a) and the fully epistatic basis detected here for shape variance and for FA (Varón-González and Navarro, 2018) suggests a genuine difference. The additive basis suggests that major genes critically influence mean shape. In contrast, the purely epistatic basis for shape variation suggests that variation is not controlled by major buffering genes as it has been sometimes proposed (e.g., Rutherford and Lindquist, 1998), but rather by a large set of interacting genes that includes previously described buffering genes (e.g., *Hsp1a* which is a hub gene in the canalization epistatic network). These genes would differently contribute to the expression of variance: some probably producing variance when others reduce it. This second view is in agreement with other experimental studies (e.g., Varón-González and Navarro, 2018) and theoretical work (e.g., Geiler-Samerotte et al., 2016, 2018).

Regulatory elements might play a particularly important role in robustness (e.g., Frankel et al., 2010; Osterwalder et al., 2018), which could contribute to the epistatic effects detected. MicroRNAs, in particular, have been proposed to impact robustness (e.g., Li et al., 2009). They are important actors of development (Hornstein and Shomron, 2006; Wu et al., 2009; Kittelmann et al., 2018) and, as regulators of genetic effects, they can explain the differential effects of the same genotypes in different genetic backgrounds (Percival et al., 2017): e.g., by regulating protein levels in the system and therefore reducing variance (Siciliano et al., 2013). Gene enhancers might be duplicated to confer robustness against mutations (Frankel et al., 2010; Osterwalder et al., 2018) and that could explain why epistatic patterns are overwhelmingly detected for canalization and developmental stability.

### Canalization and Developmental Stability: (Mostly) Similar Processes Buffering Different Sources of Variation?

Whether canalization and developmental stability correspond to the same biological process is a longstanding question. Here we show that, at least in the mice skull, they both rely on a polygenic epistatic basis and present a similar phenotypic expression, as captured by the correspondence in the patterns of shape variation among individuals (canalization) and within individuals (FA, developmental stability). Such a phenotypic resemblance is expected under the assumption of a common biological ground (Klingenberg and McIntyre, 1998; Debat et al., 2000). Yet, the similarity does not extend to the nature of the loci involved: skull canalization and developmental stability appear to involve different genes. The vast majority of the candidate genes identified were different and the network configurations (number and position of nodes) also differed.

Empirical analysis of the developmental origin of shape variation in mice (Hallgrímsson et al., 2009; Labonne et al., 2014) suggests that the patterns of covariation are variable throughout development. The similarity in the patterns of shape individual variance and shape asymmetry reported in this study suggests that variation is somehow constrained, channeled during development, along the same direction of phenotypic change. Such a channeling, that shows through the stereotyped patterns of variation produced, regardless the origin of the input variation, stochastic (asymmetric variation), or genetic and environmental (among individuals variation), *is* developmental robustness (Cheverud, 1982; Young and Badyaev, 2007; Hallgrímsson et al., 2009; Gonzalez et al., 2014). It suggests that the same processes influence developmental stability and canalization. In the same way, and keeping in mind that **E** not only depicts environmental effects but also non-additive genetic effects, the resemblance of **E** and **G** supports the view that genetic and environmental canalization rely on similar developmental processes, a contentious issue (e.g., Meiklejohn and Hartl, 2002; Siegal and Leu, 2014). Finally, the similarity of **G** and **P** suggests that **P** could be a valid surrogate of **G** in situations where **G** cannot be estimated (see discussions in Marroig and Cheverud, 2001). It suggests that the environmental component of shape variation included in **P** but not **G** is not very different from the genetic component, also pointing at a similarity of genetic and environmental canalization. Whether these results extend to other traits, models, and even mice populations is however unknown and any generalization should be considered cautiously.

In this context, the fact that the genetic networks for canalization and developmental stability are qualitatively different is difficult to interpret. If robustness is a general property of the developing skull similarly buffering individual variation and asymmetry, then we might expect similar or at least overlapping genetic architectures. Does the reported genetic difference suggest that canalization and DS are different processes? A difficulty is that the observed variation reflects both the sources of variation and the developmental processes modulating it: the difference between the two networks might thus result either from a difference in the sources of variation or from a difference in the developmental processes affecting it (or both, as distinguishing the sources of variation from the processes affecting it can be difficult). The sources of variation are likely somewhat different within and among individuals: specifically, developmental noise, originating in various phenomena, including stochastic gene expression or stochastic cellular divisions, is expected to have a higher relative importance for FA than for individual variation, which would in turn be dominated by processes with larger effects. In terms of developmental processes, genes affecting genetic or developmental noisiness [e.g., miRNA for stochastic gene expression (Schmiedel et al., 2015)]; factors involved in the regulation of the cell cycle for stochastic cellular variation (Neto-Silva et al., 2009; Debat et al., 2011; Faradji et al., 2011; Debat and Peronnet, 2013) might thus be more readily detected in the FA association mapping than for individual variation, thereby producing the observed difference between the networks. Canalization and developmental stability might thus partly differ in the processes generating and regulating some specific sources of variation among and within individuals. The channeling effect on shape variation might then occur at higher levels, or later on during morphogenesis, e.g., during tissue interactions or via the remodeling effects of mechanical demand and erase those differences (see Hallgrímsson et al., 2009).

## Conclusion

The genetic architecture of the skull shape canalization appears entirely epistatic and composed by a large proportion of craniofacial genes, as was also found for developmental stability (Varón-González and Navarro, 2018). In contrast, a large number of loci are additively associated with skull mean shape in mice (Pallares et al., 2015b) suggesting that skull shape and its variability rely on different genetic bases. The two genetic networks underlying canalization and developmental stability differ entirely in the genes involved apparently pointing at two different buffering processes. This fundamental genetic difference contrasts with the similarity in the patterns of shape variation associated with these two components of robustness. The channeling of the produced variation within the same developmental pathways could explain that despite originating from different loci these variations are expressed in a similar fashion in the skull.

Canalization and developmental stability likely involve various processes acting at various scales and times during development. Some of these processes might be specific to the type of variation considered and would thus be different between developmental stability and canalization; other processes, like bone remodeling in reaction to biomechanical demand, might jointly alter variation both among and within individuals, reshaping covariation patterns characteristic of adults structures – these would contribute to both developmental stability and canalization. Such a mix of shared and specific processes might have contributed to the diversity of results obtained in the literature comparing those two components of phenotypic robustness.

## Data Availability

The datasets analyzed for this study can be found in the Dryad repository (https://datadryad.org//resource/doi:10.5061/dryad.k543p) (Pallares et al., 2015a).

## Ethics Statement

Here we re-used the data collected for a previously published study (Pallares et al., 2015b), so no new animal manipulations have been carried for this study.

## Author Contributions

NN and CV-G designed the study. LP collected the data. NN performed the analyses. VD contributed to the discussion of the results. All authors contributed to writing the manuscript and approved its final version.

## Conflict of Interest Statement

The authors declare that the research was conducted in the absence of any commercial or financial relationships that could be construed as a potential conflict of interest.

## Footnotes

1 https://github.com/lorinanthony/MAPIT/

2 http://igraph.org

## References

[B1] Aguirre J. D. Hine E. McGuigan K. Blows M. W. (2014). Comparing G: multivariate analysis of genetic variation in multiple populations. *Heredity* 112 21–29. 10.1038/hdy.2013.12 23486079 PMC3860158

[B2] Alayan J. Ivanovski S. Farah C. S. (2007). Alveolar bone loss in T helper 1/T helper 2 cytokine-deficient mice. *J. Periodontal Res.* 42 97–103. 10.1111/j.1600-0765.2006.00920.x 17305866

[B3] Ballester M. Molist J. Lopez-Bejar M. Sánchez A. Santaló J. Folch J. M. (2004). Disruption of the mouse phospholipase C-β1 gene in a β-lactoglobulin transgenic line affects viability, growth, and fertility in mice. *Gene* 341 279–289. 10.1016/j.gene.2004.07.007 15474310

[B4] Bégin M. Roff D. A. (2004). From micro-to macroevolution through quantitative genetic variation: positive evidence from field crickets. *Evolution* 58 2287–2304. 10.1111/j.0014-3820.2004.tb01604.x 15562691

[B5] Bernstein S. E. (1980). Inherited hemolytic disease in mice: a review and update. *Lab. Anim. Sci.* 30 197–205. 6763106

[B6] Blake J. A. Eppig J. T. Kadin J. A. Richardson J. E. Smith C. L. Bult C. J. (2017). Mouse Genome Database (MGI)-2017: community knowledge resource for the laboratory mouse. *Nucleic Acids Res.* 4 D723–D729. 10.1093/nar/gkw1040 27899570 PMC5210536

[B7] Bonacich P. (1987). Power and centrality: a family of measures. *Am. J. Sociol.* 92 1170–1182. 10.1086/228631

[B8] Boyle E. A. Yang I. L. Pritchard J. K. (2017). An expanded view of complex traits: from polygenic to omnigenic. *Cell* 169 1177–1186. 10.1016/j.cell.2017.05.038 28622505 PMC5536862

[B9] Breno M. Leirs H. Van Dongen S. (2011). No relationship between canalization and developmental stability of the skull in a natural population of *Mastomys natalensis* (Rodentia: Muridae). *Biol. J. Linn. Soc.* 104 207–216. 10.1111/j.1095-8312.2011.01702.x

[B10] Breuker C. J. Patterson J. S. Klingenberg C. P. (2006). A single basis for developmental buffering of *Drosophila* wing shape. *PLoS One* 1:e7. 10.1371/journal.pone.0000007 17183701 PMC1762351

[B11] Bromiley P. A. Schunke A. C. Ragheb H. Thacker N. A. Tautz D. (2014). Semi-automatic landmark point annotation for geometric morphometrics. *Front. Zool.* 11:61 10.1186/s12983-014-0061-1

[B12] Burgio G. Baylac M. Heyer E. Montagutelli X. (2009). Genetic analysis of skull shape variation and morphological integration in the mouse using interspecific recombinant congenic strains between C57BL/6 and mice of the mus spretus species. *Evolution* 63 2668–2686. 10.1111/j.1558-5646.2009.00737.x 19490077

[B13] Cheng R. Parker C. C. Abney M. Palmer A. A. (2013). Practical considerations regarding the use of genotype and pedigree data to model relatedness in the context of genome-wide association studies. *G3* 3 1861–1867. 10.1534/g3.113.007948 23979941 PMC3789811

[B14] Cheverud J. M. (1982). Phenotypic, genetic, and environmental morphological integration in the cranium. *Evolution* 36 499–516. 10.1111/j.1558-5646.1982.tb05070.x 28568050

[B15] Churchill G. A. Doerge R. W. (1994). Empirical threshold values for quantitative trait mapping. *Genetics* 138 963–971. 7851788 10.1093/genetics/138.3.963 PMC1206241

[B16] Corty R. W. Valdar W. (2018). QTL mapping on a background of variance heterogeneity. *G3* 8 3767–3782. 10.1534/g3.118.200790 30389794 PMC6288843

[B17] Covarrubias-Pazaran G. (2016). Genome-assisted prediction of quantitative traits using the R package sommer. *PLoS One* 11:e0156744. 10.1371/journal.pone.0156744 27271781 PMC4894563

[B18] Crawford L. Zeng P. Mukherjee S. Zhou X. (2017). Detecting epistasis with the marginal epistasis test in genetic mapping studies of quantitative traits. *PLoS Genet.* 13:e1006869. 10.1371/journal.pgen.1006869 28746338 PMC5550000

[B19] Csardi G. Nepusz T. (2006). The igraph software package for complex network research. *InterJournal Complex Syst.* 1695 1–9.

[B20] De Coster G. Van Dongen S. Malaki P. Muchane M. Alcántara-Exposito A. Matheve H. (2013). Fluctuating asymmetry and environmental stress: understanding the role of trait history. *PLoS One* 8:e57966. 10.1371/journal.pone.0057966 23472123 PMC3589457

[B21] Debat V. Alibert P. David P. Paradis E. Auffray J.-C. (2000). Independence between developmental stability and canalization in the skull of the house mouse. *Proc. R. Soc. Lond. B* 267 423–430. 10.1098/rspb.2000.1017 10737397 PMC1690549

[B22] Debat V. Bloyer S. Faradji F. Gidaszewski N. Navarro N. Orozco-terWengel P. (2011). Developmental stability: a major role for cyclin G in Drosophila melanogaster. *PLoS Genet.* 7:e1002314. 10.1371/journal.pgen.1002314 21998598 PMC3188557

[B23] Debat V. David P. (2001). Mapping phenotypes: canalization, plasticity and developmental stability. *Trends Ecol. Evol.* 16 555–561. 10.1016/S0169-5347(01)02266-2

[B24] Debat V. Milton C. C. Rutherford S. Klingenberg C. P. Hoffmann A. A. (2006). Hsp90 and the quantitative variation of wing shape in *Drosophila melanogaster*. *Evolution* 60 2529–2538. 17263114

[B25] Debat V. Peronnet F. (2013) Asymmetric flies: the control of developmental noise in *Drosophila*. *Fly* 7 70–77. 10.4161/fly.23558 23519089 PMC3732334

[B26] Diez-Roux G. Banfi S. Sultan M. Geffers L. Anand S. Rozado D. (2011). A high-resolution anatomical atlas of the transcriptome in the mouse embryo. *PLoS Biol.* 9:e1000582. 10.1371/journal.pbio.1000582 21267068 PMC3022534

[B27] Dryden I. L. Koloydenko A. Zhou D. (2009). Non-Euclidean statistics for covariance matrices, with applications to diffusion tensor imaging. *Ann. Appl. Stat.* 3 1102–1123. 10.1214/09-AOAS249

[B28] Dryden I. L. Mardia K. V. (1998). *Statistical Shape Analysis.* New York, NY: John Wiley & Sons.

[B29] Dullin C. Missbach-Guentner J. Vogel W. F. Grabbe E. Alves F. (2007). Semi-automatic classification of skeletal morphology in genetically altered mice using flat-panel volume computed tomography. *PLoS Genet.* 3:e118. 10.1371/journal.pgen.0030118 17658952 PMC1934393

[B30] Faradji F. Bloyer S. Dardalhon-Cuménal D. Randsholt N. B. Peronnet F. (2011). Drosophila melanogaster Cyclin G coordinates cell growth and cell proliferation. *Cell Cycle* 10 805–818. 10.4161/cc.10.5.14959 21311225

[B31] Fisher R. A. (1935). The logic of inductive inference. *J. R. Stat. Soc.* 98 39–82. 10.2307/2342435

[B32] Flint J. Eskin E. (2012). Genome-wide association studies in mice. *Nat. Rev. Genet.* 13 807–817. 10.1038/nrg3335 23044826 PMC3625632

[B33] Frankel N. Davis G. K. Vargas D. Wang S. Payre F. Stern D. L. (2010). Phenotypic robustness conferred by apparently redundant transcriptional enhancers. *Nature* 466 490–493. 10.1038/nature09158 20512118 PMC2909378

[B34] Geiler-Samerotte K. Sartori F. M. O. Siegal M. L. (2018). Decanalizing thinking on genetic canalization. *Semin. Cell Dev. Biol.* 10.1016/j.semcdb.2018.05.008 [Epub ahead of print]. 29751086 PMC6252154

[B35] Geiler-Samerotte K. A. Zhu Y. O. Goulet B. E. Hall D. W. Siegal M. L. (2016). Selection transforms the landscape of genetic variation interacting with Hsp90. *PLoS Biol.* 14:e2000465. 10.1371/journal.pbio.2000465 27768682 PMC5074785

[B36] Gonzalez P. N. Lotto F. P. Hallgrímsson B. (2014). Canalization and developmental instability of the fetal skull in a mouse model of maternal nutritional stress. *Am. J. Phys. Anthropol.* 154 544–553. 10.1002/ajpa.22545 24888714 PMC4425270

[B37] Gonzalez P. N. Pavlicev M. Mitteroecker P. Pardo-Manuel de Villena F. Spritz R. A. Marcucio R. S. (2016). Genetic structure of phenotypic robustness in the collaborative cross mouse diallel panel. *J. Evol. Biol.* 29 1737–1751. 10.1111/jeb.12906 27234063 PMC5021570

[B38] Green R. M. Fish J. L. Young N. M. Smith F. J. Roberts B. Dolan K. (2017). Developmental nonlinearity drives phenotypic robustness. *Nat. Commun.* 8:1970. 10.1038/s41467-017-02037-7 29213092 PMC5719035

[B39] Hallgrímsson B. Green R. M. Katz D. C. Fish J. L. Bernier F. P. Roseman C. C. (2018). The developmental-genetics of canalization. *Semin. Cell Dev. Biol.* 10.1016/j.semcdb.2018.05.019 [Epub ahead of print]. 29782925 PMC6251770

[B40] Hallgrímsson B. Jamniczky H. A. Young N. M. Rolian C. Parsons T. E. Boughner J. C. (2009). Deciphering the palimpsest: studying the relationship between morphological integration and phenotypic covariation. *Evol. Biol.* 36 355–376. 10.1007/s11692-009-9076-5 23293400 PMC3537827

[B41] Hallgrímsson B. Mio W. Marcucio R. S. Spritz R. (2014). Let’s face it – Complex traits are just not that simple. *PLoS Genet.* 10:e1004724. 10.1371/journal.pgen.1004724 25375250 PMC4222688

[B42] Hallgrímsson B. Willmore K. Hall B. K. (2002). Canalization, developmental stability, and morphological integration in primate limbs. *Am. J. Phys. Anthropol.* 119 131–158. 10.1002/ajpa.10182 12653311 PMC5217179

[B43] Hernandez-Porras I. Fabbiano S. Schuhmacher A. J. Aicher A. Canamero M. Camara J. A. (2014). K-RasV14I recapitulates Noonan syndrome in mice. *Proc. Natl. Acad. Sci. U.S.A.* 111 16395–16400. 10.1073/pnas.1418126111 25359213 PMC4246321

[B44] Hornstein E. Shomron N. (2006). Canalization of development by microRNAs. *Nat. Genet.* 38:S20. 10.1038/ng1803 16736020

[B45] Houzelstein D. Bullock S. L. Lynch D. E. Grigorieva E. F. Wilson V. A. Beddington R. S. (2002). Growth and early postimplantation defects in mice deficient for the bromodomain-containing protein Brd4. *Mol. Cell. Biol.* 22 3794–3802. 10.1128/MCB.22.11.3794-3802.2002 11997514 PMC133820

[B46] Hoyt P. R. Bartholomew C. Davis A. J. Yutzey K. Gamer L. W. Potter S. S. (1997). The evil proto-oncogene is required at midgestation for neural, heart, and paraxial mesenchyme development. *Mech. Dev.* 65 55–70. 10.1016/S0925-4773(97)00057-9 9256345

[B47] Huang W. Mackay T. F. C. (2016). The genetic architecture of quantitative traits cannot be inferred from variance component analysis. *PLoS Genet.* 12:e1006421. 10.1371/journal.pgen.1006421 27812106 PMC5094750

[B48] Jones A. G. Arnold S. J. Bürger R. (2003). Stability of the G-matrix in a population experiencing pleiotropic mutation, stabilizing selection, and genetic drift. *Evolution* 57 1747–1760. 10.1111/j.0014-3820.2003.tb00583.x 14503617

[B49] Karst S. Y. Ward-Bailey P. F. Kane C. Bergstrom D. Donahue L. R. Davisson-Fahey M. T. (2010). Humpback: a new mutation on chromosome 17 causing kyphosis and abnormal muscle phenotypes. *MGI Direct Data Submission*

[B50] Kelly J. K. (2009). Connecting QTLs to the G-matrix of evolutionary quantitative genetics. *Evolution* 63 813–825. 10.1111/j.1558-5646.2008.00590.x 19087179 PMC5972393

[B51] Kittelmann S. Buffry A. D. Franke F. A. Almudi I. Yoth M. Sabaris G. (2018). Gene regulatory network architecture in different developmental contexts influences the genetic basis of morphological evolution. *PLoS Genet.* 14:e1007375. 10.1371/journal.pgen.1007375 29723190 PMC5953500

[B52] Kleinberg J. (1999). Authoritative sources in a hyperlinked environment. *J. ACM* 46 604–632. 10.1145/324133.324140

[B53] Klingenberg C. P. (2013). Cranial integration and modularity: insights into evolution and development from morphometric data. *Hystrix* 24 43–58.

[B54] Klingenberg C. P. (2015). Analyzing fluctuating asymmetry with geometric morphometrics: concepts, methods, and applications. *Symmetry* 7 843–934. 10.3390/sym7020843

[B55] Klingenberg C. P. Barluenga M. Meyer A. (2002). Shape analysis of symmetric structures: quantifying variation among individuals and asymmetry. *Evolution* 56 1909–1920. 10.1111/j.0014-3820.2002.tb00117.x 12449478

[B56] Klingenberg C. P. Debat V. Roff D. A. (2010). Quantitative genetics of shape in cricket wings: developmental integration in a functional structure. *Evolution* 64 2935–2951. 10.1111/j.1558-5646.2010.01030.x 20482613

[B57] Klingenberg C. P. Leamy L. (2001). Quantitative genetics of geometric shape in the mouse mandible. *Evolution* 55 2342–2352. 10.1111/j.0014-3820.2001.tb00747.x 11794792

[B58] Klingenberg C. P. McIntyre G. S. (1998). Geometric morphometrics of developmental instability: analyzing patterns of fluctuating asymmetry with procrustes methods. *Evolution* 52 1363–1375. 10.1111/j.1558-5646.1998.tb02018.x 28565401

[B59] Klingenberg C. P. Monteiro L. R. (2005). Distances and directions in multidimensional shape spaces: implications for morphometric applications. *Syst. Biol.* 54 678–688. 10.1080/10635150590947258 16126663

[B60] Kolsch U. Arndt B. Reinhold D. Lindquist J. A. Juling N. Kliche S. (2006). Normal T-cell development and immune functions in TRIM-deficient mice. *Mol. Cell. Biol.* 2006:9. 10.1128/MCB.26.9.3639-3648.2006 16612002 PMC1447406

[B61] Krzanowski W. J. (1979). Between-groups comparison of principal components. *J. Am. Stat. Assoc.* 74 703–707. 10.1080/01621459.1979.10481674

[B62] Labonne G. Navarro N. Laffont R. Chateau-Smith C. Montuire S. (2014). Developmental integration in a functional unit: deciphering processes from adult dental morphology. *Evol. Dev.* 16 224–232. 10.1111/ede.12085 25040671

[B63] Lande R. (1979). Quantitative genetic analysis of multivariate evolution, applied to brain:body size allometry. *Evolution* 33 402–416. 10.1111/j.1558-5646.1979.tb04694.x 28568194

[B64] Leamy L. J. Klingenberg C. P. (2005). The genetics and evolution of fluctuating asymmetry. *Annu. Rev. Ecol. Evol. Syst.* 36 1–21. 10.1146/annurev.ecolsys.36.102003.152640

[B65] Leamy L. J. Klingenberg C. P. Sherratt E. Wolf J. B. Cheverud J. M. (2015). The genetic architecture of fluctuating asymmetry of mandible size and shape in a population of mice: another look. *Symmetry* 7 146–163. 10.3390/sym7010146

[B66] Li X. Cassidy J. J. Reinke C. A. Fischboeck S. Carthew R. W. (2009). A MicroRNA imparts robustness against environmental fluctuation during development. *Cell* 137 273–282. 10.1016/j.cell.2009.01.058 19379693 PMC2674871

[B67] Listgarden J. Lippert C. Kadie C. M. Davidson R. I. Eskin E. Heckerman D. (2012). Improved linear mixed models for genome-wide association studies. *Nat. Methods* 9 525–526. 10.1038/nmeth.2037 22669648 PMC3597090

[B68] Mackay T. F. C. (2014). Epistasis and quantitative traits: using model organisms to study gene-gene interactions. *Nat. Rev. Genet.* 15 22–33. 10.1038/nrg3627 24296533 PMC3918431

[B69] Maga A. M. Navarro N. Cunningham M. L. Cox T. C. (2015). Quantitative trait loci affecting the 3D skull shape and size in mouse and prioritization of candidate genes in-silico. *Front. Physiol.* 6:92. 10.3389/fphys.2015.00092 25859222 PMC4374467

[B70] Mardia K. V. Bookstein F. Moreton I. J. (2000). Statistical assessment of bilateral symmetry of shapes. *Biometrika* 87 285–300. 10.1016/j.joen.2017.01.012 28377148

[B71] Marroig G. Cheverud J. M. (2001). A comparison of phenotypic variation and covariation patterns and the role of phylogeny, ecology, and ontogeny during cranial evolution of new world monkeys. *Evolution* 55 2576–2600. 10.1111/j.0014-3820.2001.tb00770.x 11831671

[B72] Martínez-Espinosa P. L. Yang C. Gonzalez-Perez V. Xia X.-M. Lingle C. J. (2014). Knockout of the BK β2 subunit abolishes inactivation of BK currents in mouse adrenal chromaffin cells and results in slow-wave burst activity. *J. Gen. Physiol.* 144 275. 10.1085/jgp.201411253 25267913 PMC4178941

[B73] Masuda T. Kai N. Sakuma C. Kobayashi K. Koga H. Yaginuma H. (2009). Laser capture microdissection and cDNA array analysis for identification of mouse KIAA/FLJ genes differently expressed in the embryonic dorsal spinal cord. *Brain Res.* 1249 61–67. 10.1016/j.brainres.2008.10.028 19026994

[B74] Meiklejohn C. D. Hartl D. L. (2002). A single mode of canalization. *Trends Ecol. Evol.* 17 468–473. 10.1016/S0169-5347(02)02596-X 8844166

[B75] Melo D. Garcia G. Hubbe A. Assis A. P. Marroig G. (2016). EvolQG – An R package for evolutionary quantitative genetics. *F1000Res.* 4:925. 10.12688/f1000research.7082.2 27785352 PMC5022708

[B76] Meyer K. (2009). Factor-analytic models for genotype x environment type problems and structured covariance matrices. *Genet. Sel. Evol.* 41:21. 10.1186/1297-9686-41-21 19284520 PMC2674411

[B77] Miettinen P. J. Chin J. R. Shum L. Slavkin H. C. Shuler C. F. Derynck R. (1999). Epidermal growth factor receptor function is necessary for normal craniofacial development and palate closure. *Nat. Genet.* 22 69–73. 10.1038/8773 10319864

[B78] Mitteroecker P. Bookstein F. (2009). The ontogenetic trajectory of the phenotypic covariance matrix, with examples from craniofacial shape in rats and humans. *Evolution* 63 727–737. 10.1111/j.1558-5646.2008.00587.x 19087182

[B79] Moon A. M. Stauffer A. M. Schwindinger W. F. Sheridan K. Firment A. Robishaw J. D. (2014). Disruption of G-protein γ5 subtype causes embryonic lethality in mice. *PLoS One* 9:e90970. 10.1371/journal.pone.0090970 24599258 PMC3944967

[B80] Navarro N. Maga A. M. (2016). Does 3D phenotyping yield substantial insights in the genetics of the mouse mandible shape? *G3* 6 1153–1163. 10.1534/g3.115.024372 26921296 PMC4856069

[B81] Neto-Silva R. M. Wells B. S. Johnston L. A. (2009). Mechanisms of growth and homeostasis in the Drosophila wing. *Annu. Rev. Cell Dev.* 25 197–220. 10.1146/annurev.cellbio.24.110707.175242 PMC2760035 19575645

[B82] Nicod J. Davies R. W. Cai N. Hassett C. Goodstadt L. Cosgrove C. (2016). Genome-wide association of multiple complex traits in outbred mice by ultra-low-coverage sequencing. *Nat. Genet.* 48 912–918. 10.1038/ng.3595 27376238 PMC4966644

[B83] Osterwalder M. Barozzi I. Tissières V. Fukuda-Yuzawa Y. Mannion B. J. Afzal S. Y. (2018). Enhancer redundancy provides phenotypic robustness in mammalian development. *Nature* 554:239. 10.1038/nature25461 29420474 PMC5808607

[B84] Pallares L. F. Carbonetto P. Gopalakrishnan S. Parker C. C. Ackert-Bicknell C. L. Palmer A. A. (2015a). Data from: mapping of craniofacial traits in outbred mice identifies major developmental genes involved in shape determination. *PLoS Genet.* 11:e1005607. 10.1371/journal.pgen.1005607 26523602 PMC4629907

[B85] Pallares L. F. Carbonetto P. Gopalakrishnan S. Parker C. C. Ackert-Bicknell C. L. Palmer A. A. (2015b). Mapping of craniofacial traits in outbred mice identifies major developmental genes involved in shape determination. *PLoS Genet.* 11:e1005607. 10.1371/journal.pgen.1005607 26523602 PMC4629907

[B86] Pallares L. F. Harr B. Turner L. M. Tautz D. (2014). Use of a natural hybrid zone for genomewide association mapping of craniofacial traits in the house mouse. *Mol. Ecol.* 23 5756–5770. 10.1111/mec.12968 25319559

[B87] Pallares L. F. Turner L. M. Tautz D. (2016). Craniofacial shape transition across the house mouse hybrid zone: implications for the genetic architecture and evolution of between-species differences. *Dev. Genes Evol.* 226 173–186. 10.1007/s00427-016-0550-7 27216933 PMC4896993

[B88] Palmer A. R. Strobeck C. (1986). Fluctuating asymmetry: measurement, analysis, patterns. *Annu. Rev. Ecol. Syst.* 17 391–421. 10.1146/annurev.es.17.110186.002135

[B89] Parker C. C. Carbonetto P. Sokoloff D. D. Park Y. J. Abney M. Palmer A. A. (2014). High-resolution genetic mapping of complex traits from a combined analysis of F2 and advanced intercross mice. *Genetics* 198 103–116. 10.1534/genetics.114.167056 25236452 PMC4174923

[B90] Percival C. J. Marangoni P. Tapaltsyan V. Klein O. Hallgrímsson B. (2017). The interaction of genetic background and mutational effects in regulation of mouse craniofacial shape. *G3* 7 1439–1450. 10.1534/g3.117.040659 28280213 PMC5427488

[B91] Perkowski J. J. Murphy G. G. (2011). Deletion of the mouse homolog of KCNAB2, a gene linked to monosomy 1p36, results in associative memory impairments and amygdala hyperexcitability. *J. Neurosci.* 31 46–54. 10.1523/JNEUROSCI.2634-10.2011 21209188 PMC3078585

[B92] Piazza R. Magistroni V. Redaelli S. Mauri M. Massimino L. Sessa A. (2018). SETBP1 induces transcription of a netwrok of development genes by acting as an epigenetic hub. *Nat. Commun.* 9:2192. 10.1038/s41467-018-04462-8 29875417 PMC5989213

[B93] R Core Team (2013). *R: A Language and Environment for Statistical Computing.* Vienna: R Foundation for Statistical Computing.

[B94] Roff D. A. Prokkola J. M. Krams I. Rantala M. J. (2012). There is more than one way to skin a G matrix. *J. Evol. Biol.* 25 1113–1126. 10.1111/j.1420-9101.2012.02500.x 22487403

[B95] Rönnegård L. Valdar W. (2011). Detecting major genetic loci controlling phenotypic variability in experimental crosses. *Genetics* 188 435–447. 10.1534/genetics.111.127068 21467569 PMC3122324

[B96] Runcie D. E. Mukherjee S. (2013). Dissecting high-dimensional phenotypes with bayesian sparse factor analysis of genetic covariance matrices. *Genetics* 194 753–767. 10.1534/genetics.113.151217 23636737 PMC3697978

[B97] Rutherford S. L. Lindquist S. (1998). Hsp90 as a capacitor for morphological evolution. *Nature* 396 336–342. 10.1038/24550 9845070

[B98] Schluter D. (1996). Adaptive radiation along genetic lines of least resistance. *Evolution* 50 1766–1774. 10.1111/j.1558-5646.1996.tb03563.x 28565589

[B99] Schmiedel J. M. Klemm S. L. Zheng Y. Sahay A. Blüthgen N. Marks D. S. (2015). MicroRNA control of protein expression noise. *Science* 348 128–132. 10.1126/science.aaa1738 25838385

[B100] Schoenebeck J. J. Ostrander E. A. (2013). The genetic of canince skull shape variation. *Genetics* 193 317–325. 10.1534/genetics.112.145284 23396475 PMC3567726

[B101] Shen-Li H. O’Hagan R. Hou H. J. Horner J. W. II Lee H.-W. DePinho R. A. (2000). Essential role for Max in early embryonic growth and development. *Genes Dev.* 14 17–22. 10640271 PMC316346

[B102] Siciliano V. Garzilli I. Fracassi C. Criscuolo S. Ventre S. di Bernardo D. (2013). MiRNAs confer phenotypic robustness to gene networks by supressing biological noise. *Nat. Commun.* 4:2364. 10.1038/ncomms3364 24077216 PMC3836244

[B103] Siegal M. L. Leu J.-Y. (2014). On the nature and evolutionary impact of phenotypic robustness mechanisms. *Annu. Rev. Ecol. Evol. Syst.* 45 495–517. 10.1146/annurev-ecolsys-120213-091705 26034410 PMC4448758

[B104] Speed D. Balding D. J. (2015). Relatedness in the post-genomic era: is it still useful? *Nat. Rev. Genet.* 16 33–44. 10.1038/nrg3821 25404112

[B105] Takahashi K. H. Rako L. Takano-Shimizu T. Hoffmann A. A. Lee S. F. (2010). Effect of small *Hsp* genes on developmental stability and microenvironmental canalization. *BMC Evol. Biol.* 10:284. 10.1186/1471-2148-10-284 20846409 PMC2949873

[B106] Teplitsky C. Robinson M. R. Merilä J. (2014). “Evolutionary potential and constraints in wild populations,” in *Quantitative Genetics in Wild Populations*, eds Charmantier A. Garant D. Kruuk L. E. B. (Oxford: Oxford University Press), 190–208.

[B107] Uller T. Moczek A. P. Watson R. A. Brakefield P. M. Laland K. N. (2018). Developmental bias and evolution: a regulatory network perspective. *Genetics* 209 949–966. 10.1534/genetics.118.300995 30049818 PMC6063245

[B108] Van Dongen S. (1998). How repeatable is the estimation of developmental stability by fluctuating asymmetry? *Proc. R. Soc. BBiol. Sci.* 265 1423–1427. 10.1098/rspb.1998.0452

[B109] Varón-González C. Navarro N. (2018). Epistasis regulates the developmental stability of the mouse craniofacial shape. *Heredity* 10.1038/s41437-018-0140-8 30209292 PMC6461946

[B110] Visel A. Thaller C. Eichele G. (2004). GenePaint.org: an atlas of gene expression patterns in the mouse embryo. *Nucleic Acids Res.* 32 D552–D556. 14681479 10.1093/nar/gkh029 PMC308763

[B111] Wada T. Nakashima T. Oliveira-Dos-Santos A. J. Gasser J. Hara H. Schett G. (2005). The molecular scaffold Gab2 is a crucial component of RANK signaling and osteoclastogenesis. *Nat. Med.* 11 394–399. 10.1038/nm1203 15750601

[B112] Wagner G. P. (1984). On the eigenvalue distribution of genetic and phenotypic dispersion matrices: evidence for a nonrandom organization of quantitative character variation. *J. Math. Biol.* 21 77–95. 10.1007/BF00275224

[B113] Wagner G. P. Booth G. Bagheri-Chaichian H. (1997). A population genetic theory of canalization. *Evolution* 51 329–347. 10.1111/j.1558-5646.1997.tb02420.x 28565347

[B114] Wei P. Satoh T. Edamatsu H. Aiba A. Setsu T. Terashima T. (2007). Defective vascular morphogenesis and mid-gestation embryonic death in mice lacking RA-GEF-1. *Biochem. Biophys. Res. Commun.* 363 106–112. 10.1016/j.bbrc.2007.08.149 17826737

[B115] Weinberg S. M. Cornell R. Leslie E. J. (2018). Craniofacial genetics: where have we been and where are we going? *PLoS Genet.* 14:e1007438. 10.1371/journal.pgen.1007438 29927928 PMC6013018

[B116] Willmore K. Zelditch M. L. Young N. Ah-Seng A. Lozanoff S. Hallgrímsson B. (2006). Canalization and developmental stability in the Brachyrrhine mouse. *J. Anat.* 208 361–372. 10.1111/j.1469-7580.2006.00527.x 16533318 PMC2100242

[B117] Wolf J. B. Leamy L. J. Routman E. J. Cheverud J. M. (2005). Epistatic pleiotropy and the genetic architecture of covariation within early and late-developing skull trait complexes in mice. *Genetics* 171 683–694. 10.1534/genetics.104.038885 16020793 PMC1456798

[B118] Wolf J. B. Pomp D. Eisen E. J. Cheverud J. M. Leamy L. J. (2006). The contribution of epistatic pleiotropy to the genetic architecture of covariation among polygenic traits in mice. *Evol. Dev.* 8 468–476. 10.1111/j.1525-142X.2006.00120.x 16925682

[B119] Won J. Marin de Evsikova C. Smith R. S. Hicks W. L. Edwards M. M. Longo-Guess C. (2011). NPHP4 is necessary for normal photoreceptor ribbon synapse maintenance and outer segment formation, and for sperm development. *Hum. Mol. Genet.* 20 482–496. 10.1093/hmg/ddq494 21078623 PMC3016909

[B120] Wu C.-I. Shen Y. Tang T. (2009). Evolution under canalization and the dual roles of microRNAs – A hypothesis. *Genome Res.* 19 734–743. 10.1101/gr.084640.108 19411598 PMC3647535

[B121] Yates A. Akanni W. Amode M. R. Barrell D. Billis K. Carvalho-Silva D. (2016). Ensembl 2016. *Nucleic Acids Res.* 44 D710–D716. 10.1093/nar/gkv1157 26687719 PMC4702834

[B122] Young R. Maga A. M. (2015). Performance of single and multi-atlas based automated landmarking methods compared to expert annotations in volumetric microCT datasets of mouse mandibles. *Front. Zool.* 12:33. 10.1186/s12983-015-0127-8 26628903 PMC4666065

[B123] Young R. L. Badyaev A. V. (2007). Evolution of ontogeny: linking epigenetic remodeling and genetic adaptation in skeletal structures. *Integr. Comp. Biol.* 47 234–244. 10.1093/icb/icm025 21672834

[B124] Zakharov V. M. (1992). Population phenogenetics: analysis of developmental stability in natural populations. *Acta Zool. Fenn.* 191 7–30.

